# Intra-abdominal hypertension in patients with sellar region tumors

**DOI:** 10.1186/2110-5820-2-S1-S2

**Published:** 2012-07-05

**Authors:** Konstantin A Popugaev, Ivan A Savin, Andrew U Lubnin, Alexander S Goriachev, Boris A Kadashev, Pavel L Kalinin, Andrew V Oshorov, Alexander A Polupan, Ekaterina U Sokolova, Maxim A Kutin, Valeriy I Lukianov

**Affiliations:** 1 Department of Neurological Intensive Care Unit (NICU), Burdenko Neurosurgical Research Institute, 16 4th Tverskaya-Yamskaya, Moscow, 125047, Russia

**Keywords:** intra-abdominal pressure, intra-abdominal hypertension, abdominal compartment syndrome, epidural anesthesia, neurocritical care, sellar region tumor, ileus, postoperative complication.

## Abstract

**Background:**

Data on intra-abdominal hypertension [IAH] and secondary abdominal compartment syndrome [ACS] due to neurological insults are limited.

**Methods:**

This was a prospective observational study conducted between January 2010 and January 2011 in the neurological ICU [NICU]. Forty-one consecutive patients with sellar region tumors [SRT] were enrolled into the study. If conservative therapy was ineffective in patients with ACS, thoracic epidural anesthesia [EA] was performed. Primary endpoint was defined as the efficacy of conservative treatment and EA in patients with IAH and ACS; secondary endpoint, the influence of IAH and ACS on outcomes.

**Results:**

Of the 41 patients, 13 (31.7%) had normal intra-abdominal pressure and 28 (68.3%) developed IAH, of whom 9 (22%) had ACS (group II). On average, IAH developed on the second postoperative day, while ACS, between the third and the fifth day. Multiple organ dysfunction developed in 3 (23.1%) patients of group I and in 23 (82%) patients of group II (*p *= 0.0003). Ileus due to gastrointestinal dysmotility was present in 6 (46.2%) patients of group I and in all patients of group II (*p *= 0.0001). Significant risk factors for ileus were diencephalon dysfunction (whole group - in 33 patients (80.5%); group I - in 6 patients (46.2%); group II - in 27 patients (96.4%), *p *= 0.0002) and sepsis (whole group - in 8 patients (19.5%); group I - no cases; group II - in 8 patients (28.6%), *p *= 0.03). Conservative treatment was effective in the majority of patients (78.9%) with IAH and only in 3 (33%) patients with ACS. Thoracic EA was performed in four patients with ACS with success. Length of stay in the NICU was 6.5 ± 4.6 days in group I and 24.1 ± 25.7 (*p *= 0.02) days in group II. Five out of nine (55.6%) patients with ACS died. None of these patients received EA. All patients with EA had favorable outcomes.

**Conclusion:**

The development of IAH is common after SRT surgery. If conservative treatment is ineffective, EA can be considered in patients with secondary ACS. Further studies are warranted.

## Introduction

Intra-abdominal hypertension [IAH] is associated with increased morbidity and mortality in critically ill patients [1-3]. Abdominal compartment syndrome [ACS] leads to multiple organ dysfunction [MOD] and carries a high mortality [4,5]. Ileus is considered as a contributing factor leading to IAH [1]. Patients with recent removal of sellar region tumors [SRT] represent a special neurocritical care population because they have an increased risk of postoperative ileus [4].

The ideal management for secondary ACS has not yet been well defined [6,7]. The evidence-based therapeutic options are scarce [5,8]. The consensus conference on IAH and ACS recommends to maintain an abdominal perfusion pressure [APP] above 50 to 60 mmHg (grade 1C), to use a brief trial of neuromuscular blockage (grade 2C), to avoid elevation of the head of the bed above 30° (grade 2C), and to use hypertonic crystalloid and colloid-based resuscitation fluids (grade 1C) [5]. The benefit of analgesia/sedation, prokinetic motility agents, and nasogastric/colonic decompression is unclear [5]. Intra-abdominal pressure [IAP] should be decreased before it reaches the threshold that will lead to the development of irreversible MOD. Urgent abdominal decompression is recommended in patients in whom medical treatment for ACS has failed [9]. In spite of the relative safety and absolute efficacy of abdominal decompression, this operation has inherent risks and morbidity [8]. Prolonged thoracic epidural anesthesia [EA] has been shown to be effective in the reduction of IAP in patients with primary ACS [10]. To date, there are no data on the efficacy of EA in patients with secondary ACS. The purpose of the study was (1) to investigate the incidence of raised IAP in the postoperative period in SRT patients, (2) to evaluate the efficacy of conservative treatment in IAH and (3) to evaluate the efficacy of EA as a treatment option for ACS.

## Methods

### Study patients and definitions

Between January 2010 and January 2011, a prospective observational study was conducted in the 38-bed neurological intensive care unit [NICU] of Burdenko Neurosurgical Research Institute, Moscow, Russia. Inclusion criteria were: (1) adult patients, (2) SRT, and (3) a complicated postoperative period. Exclusion criteria were: (1) deep coma, (2) phimosis or contraindication for bladder pressure measurement, and (3) a contracted bladder. Patients were selected consecutively. All consecutive patients who met the inclusion criteria were included into the study. The study was conducted in accordance with the study protocol, the Declaration of Helsinki, and applicable regulatory requirements. The institutional review board and the local institutional ethics committee approved the protocol. Informed consent was obtained from each patient before inclusion.

A complicated postoperative period was recognized if the patient had an unstable neurological status or a new organ dysfunction. The clinical picture in SRT patients is typically a combination of altered level of consciousness, water and electrolyte disturbances (fluid balance dysregulation with hyper- or hyponatremia in the absence of iatrogenic causes, such as inadequate use of desmopressin, vaptans (V_2_-receptor antagonists), or hyperosmotic solutions), and at least one new organ dysfunction/failure as defined by a sequential organ failure assessment [SOFA] subscore equal to or above 3 [4]. This entity has been defined previously as diencephalon dysfunction [DD].

IAH is defined as a sustained elevation of IAP equal to or above 12 mmHg, and ACS is defined as a sustained increase in IAP over 20 mmHg that is associated with a new organ dysfunction/failure [2]. The APP is calculated as mean arterial pressure minus IAP.

One of the causes of IAH and secondary ACS is gastrointestinal dysmotility resulting in ascites and ileus. Ileus has been classified into three forms: (1) impaired gastric emptying, (2) impaired intestinal/colonic emptying, (3) or both. Impaired gastric emptying is defined as a gastric residual volume above 400 ml per day (gastrostasis) with preserved intestinal peristalsis and defecation. Impaired intestinal/colonic emptying is defined as preserved gastric emptying with depressed intestinal peristalsis and constipation. Gastrostasis, depressed intestinal peristalsis, and constipation comprise the combined form of ileus. Outcomes were evaluated with the Glasgow Outcome Scale [GOS] (Table 1).

**Table 1:** Glasgow Outcome Scale

GOS	Outcome	Functional outcome
1	Dead	Unfavorable
2	Vegetative state	
3	Severely disabled (conscious but requires others for daily support)	
4	Moderately disabled (independent but disabled)	Favorable
5	Good recovery (resumed most normal activity)	

### Patient management

Severity scores (acute physiology and chronic health evaluation [APACHE]-II and SOFA) were recorded in all patients admitted to NICU. Routine CT imaging of the brain was performed immediately postsurgery. Most patients were ventilated, and crystalloids or colloids and sympathomimetics were started early when indicated and to maintain perfusion pressure [4].

All patients received polyhormonal replacement therapy with glucocorticoids (hydrocortisone 150 to 200 mg/day which gradually decrease to 30 mg/day, orally) and thyroid hormones (L-thyroxin 1 to 3 μg/kg/day, orally or IV). Central diabetes insipidus was corrected with desmopressin.

All physiological, clinical, and biochemical data were collected. All patients were assessed with APACHE-II and SOFA scores.

### IAP, APP measurement, and IAH management

IAP was measured every 6 h from the second postoperative day and during ICU stay, or until the 28th postoperative day. Measurements were carried out via the bladder with an installation volume of 25 ml sterile saline. Urinary catheter was connected to a Philips MP60 (Eindhoven, The Netherlands) monitor via a standard pressure transducer [11]. The APP was registered simultaneously.

All the patients underwent ultrasound investigation in order to rule out ascites. Patients were screened for IAH risk factors according to an international consensus [2]. Risk factors for ileus were considered to be either independent or related to the presence of DD. Independent risk factors are the use of narcotic analgetics, sympathomimetics, positive fluid balance, sepsis, and hypokalemia.

Conservative treatment of IAH was started immediately in accordance to the IAH/ACS medical management algorithm [5,12]. If conservative treatment was ineffective in patients with ACS for longer than a 24-h period, thoracic EA was performed via an 18-gauge epidural catheter that was inserted at the level of T8 to T9 through a Touhy needle and advanced up to 5 to 6 cm in a cephalad direction. After confirmation of catheter position, a 0.25% bupivacaine infusion at 7 to 8 ml/h was initiated and titrated. The EA remained in place for 3 days. Sepsis was a contraindication for EA. According to our department protocol, treatment of IAH was considered effective if IAP normalized within 72 h; and treatment of ACS was considered effective if IAP normalized within 24 h.

### Statistical methods

The primary endpoint was defined as the efficacy of conservative treatment and EA to lower IAP in patients with IAH and ACS. The secondary endpoint was defined as the influence of IAP and ACS on the outcome. Descriptive analyses were performed for all study variables. Data are presented as mean and standard deviation. We used variance analysis, *χ*^2 ^(Pearson's and maximum likelihood chi-square), and Fisher's exact test in the analysis of contingency tables. We also calculated the relative risk when appropriate. A *p *value of < 0.05 was defined as a significant difference. For this purpose, we used program Statistica v6 (StatSoft Inc., Tulsa, OK, USA).

## Results

### Patient characteristics and patient groups

**Figure 1:**
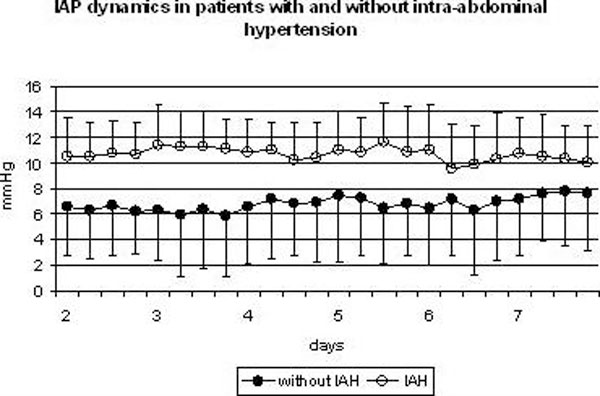
**Mean IAP in groups I and II during the first seven days of postoperative period**.

Five hundred and seventy-eight SRT patients underwent tumor removal during the study period. Five hundred and thirty-four patients were excluded because they had a normal postoperative period; another three patients were excluded because they developed deep coma on the second postoperative day. The remaining 41 patients were enrolled. The IAP was normal in 13 patients (group I). IAH developed in 28 patients (group II, Figure 1). Baseline APACHE-II and SOFA scores for group II patients indicate that their condition was more severe at the time of NICU admission (Table 2).

**Table 2:** Characteristics of group I and group II

	Whole group	Group I (no IAH)	Group II (IAH)	*p *Value (significance of difference between groups I and II)
Number (patients)	41	13	28	
Sex (male/female)	19:22	8:5	11:17	0.2
Age (mean ± SD)	48.3 ± 15	46.3 ± 17.7	49.3 ± 13.8	0.8
APACHE-II (mean ± SD)	13 ± 5.6	9.1 ± 6.1	14.9 ± 4.4	0.0004*
SOFA score (mean ± SD)	2.5 ± 1.8	1.9 ± 2.3	2.7 ± 1.6	0.2
Neurosurgical pathology (patients)				
Pituitary adenoma	18	6	12	0.8
Craniopharyngioma	11	1	10	0.06
Sellar region meningioma	8	3	5	0.7
III ventricular glioma	3	2	1	0.2
III ventricular ganglioma	1	1	0	0.1
Admission value of IAP (mmHg, mean ± SD)	9.2 ± 4.0	6.5 ± 3.1	10.6 ± 3.9	0.003*
Mean value of IAP mmHg (mean ± SD)	9.3 ± 3.1	6.4 ± 2.0	10.7 ± 2.5	0.000003*
Maximal value of IAP (mmHg, mean ± SD)	15.2 ± 6.2	8.9 ± 2.3	18.6 ± 5.6	0.002*
Minimal value of APP (mmHg, mean ± SD)	79.0 ± 9.0	86.5 ± 5.8	75.6 ± 8.2	0.0001*
Risk factors for IAH (patients (%))				
Positive fluid balance	7 (17.1)	1 (7.7)	6 (21.4)	0.3
Acidosis	2 (4.9	0 (0)	2 (7.1)	0.3
Hypothermia	4 (9.8)	1 (7.7)	3 (10.7)	0.7
Coagulopathy	6 (14.6)	1 (7.7)	5 (17.9)	0.4
MOD	26 (63.4)	3 (23.1)	23 (82)	0.0003*
MV	24 (58.5)	6 (46.2)	18 (64.3)	0.3
Ileus	34 (82.9)	6 (46.2)	28 (100)	0.0001*
Type of ileus (patients (%))				
Impaired gastric emptying	-	-	-	
Impaired intestinal and colonic emptying	21 (51.2)	4 (30.8)	17 (60.7)	0.07
Combined form	13 (31.7)	2 (15.4)	11 (39.3)	0.1
Onset time of ileus (postoperative day)	2.3 ± 1.4	1.7 ± 0.5	2.4 ± 1.5	0.17
Duration of ileus (days)	10.6 ± 7.6	4.3 ± 1.0	11.9 ± 7.7	0.01*
Risk factors of ileus (patients (%))				
DD	33 (80.5)	6 (46.2)	27 (96.4)	0.0002*
Independent risk factors (patients (%))				
Narcotics	2 (4.9)	0 (0)	2 (7.1)	0.3
Sympathomimetics	7 (17.1)	5 (38.5)	2 (7.1)	0.01*
Positive fluid balance	7 (17.1)	1 (7.7)	6 (21.4)	0.3
Sepsis	8 (19.5)	0 (0)	8 (28.6)	0.03*
Hypokalemia, hypomagnesaemia	28 (68.3)	8 (61.5)	20 (71.4)	0.5
LOS (days, mean ± SD)	18.5 ± 22.8	6.5 ± 4.6	24.1 ± 25.7	0.02*
Outcome (patients (%))				
Favorable outcome GOS 4	26 (63.4)	11 (85)	15 (54)	0.06
Unfavorable outcome	15 (36.6)	2 (15)	13 (46)	0.06
GOS 2,3	4 (9.8)	0 (0)	4 (14)	
GOS 1	11 (26.8)	2 (15)	9 (32)	

*Significant difference. IAH, intra-abdominal hypertension; SD, standard deviation; APACHE-II, acute physiology and chronic health evaluation, and SOFA, sequential organ failure assessment, scores on ICU admission; IAP, intra-abdominal pressure; APP, abdominal perfusion pressure; MOD, multiple organ dysfunction; MV, mechanical ventilation; GOS, Glasgow Outcome Scale; LOS, length of stay.

### IAP and APP

The majority of patients were admitted to NICU without IAH. In group I, IAP was significantly lower than in group II, 6.5 ± 3.1 mmHg versus 10.6 ± 3.9 mmHg (*p *= 0.003, Table 2). In group I, the duration of IAP monitoring was 5.6 ± 4.8 days; in group II, it was 14.9 ± 8.5 days. The IAP was significantly higher in group II from the second to the sixth postoperative day. Maximal IAP was 8.9 ± 2.3 mmHg in group I and 18.6 ± 5.6 mmHg in group II. In group II, maximal IAP developed on 2.5 ± 0.9 postoperative day; in group I, significantly later - on 6.2 ± 0.9 postoperative day (*p *= 0.01). ACS developed in nine patients. On average, IAH developed on the second postoperative day, while ACS, between the third and the fifth day. Minimal APP was normal in both groups, however, it was significantly higher in group I - 86.5 ± 5.8 mmHg versus 75.6 ± 8.2 mmHg in group II (*p *= 0.0001).

### Causes of IAH and types of ileus

None of the patients developed ascites. Ileus was the most common risk factor for IAH in both groups, 46.2% and 100%, respectively. MOD and the need for mechanical ventilation [MV] for more than 24 h occurred also more frequently in group II (Table 2). Ileus developed within the first two postoperative days, and its onset time did not differ between groups (*p *= 0.17). Duration of ileus was significantly longer in group II (*p *= 0.01). There were no cases with isolated impaired gastric emptying. The impaired intestinal and colonic emptying was diagnosed in 4 patients of group I and in 17 patients of group II (*p *= 0.07). An interesting observation was that ileus developed prior to IAH, but it lasted longer.

A major cause of ileus was DD. DD developed in 46.2% cases of group I and in 96.4% cases of group II. Among the list of independent risk factors, electrolyte disorders and sympathomimetics were common in both groups (Table 2).

### Diencephalon dysfunction

DD significantly increased the risk for IAH development (relative risk 6.56, 95% confidence interval 1.04 to 16.46).

### Therapy of IAH and ACS

Conservative treatment was effective in 15 patients out of 19 (79%) with IAH, but without ACS and in 3 patients out of 9 (33%) with ACS. EA was performed in four cases with ACS. In all these cases, EA effectively normalized IAP rapidly after bupivacaine infusion. No complications were associated with EA. In three ACS cases, conservative treatment was effective, and in two patients with sepsis and ACS, EA was contraindicated.

### Outcomes

Eleven patients (85%) of group I had a favorable outcome, and only two patients (15%) died due to brain edema and herniation early in the postoperative period. In group II, a favorable outcome was observed in 15 patients (54%), while 4 patients (14%) had severe disability, and 9 patients died (32%). Causes of death were: sepsis (*n *= 3), meningitis (*n *= 1), combination of sepsis and meningitis (*n *= 2), brain edema and herniation (*n *= 1), acromegalic cardiomyopathy (*n *= 1), and pulmonary thromboembolism (*n *= 1). An absolute risk of having a poorer neurologic recovery (any other below GOS 5) in patients with IAH was 0.89, with a 95% confidence interval of 0.77 to 1.0. The length of stay [LOS] in the NICU was significantly longer in patients with IAH (*p *= 0.02, Table 2).

**Figure 2:**
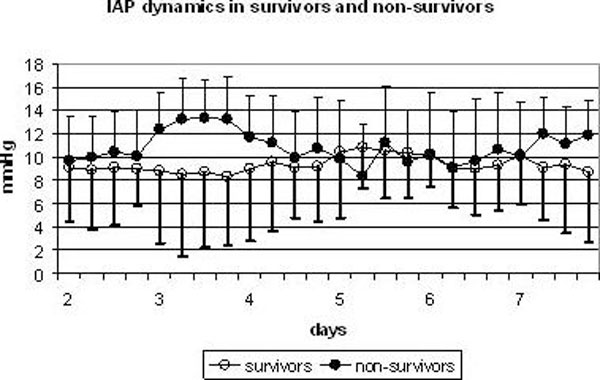
**Mean IAP in survivors and non-survivors during the first seven days of postoperative period**.

IAH developed in 19 (63.3%) survivors and in 9 (81.8%) non-survivors (*p *= 0.3). ACS developed in 13.3% of survivors and in 45.5% of non-survivors (*p *= 0.03) (Table 3). The evolution of IAP in survivors and non-survivors during the first seven days of the postoperative period is shown in Figure 2.

**Table 3:** Characteristics of survivors and non-survivors

	Survivors	Non-survivors	*p *Value
Number (patients)	30	11	
Sex (male/female)	12:18	7:4	0.17
Age (mean ± SD)	46.6 ± 15.9	53.0 ± 11.3	0.8
APACHE-II (mean ± SD)	11.0 ± 4.5	18.6 ± 4.6	0.05
SOFA score (mean ± SD)	1.9 ± 1.4	4.1 ± 2.1	0.04*
Admission value of IAP (mmHg, mean ± SD)	9.1 ± 3.9	9.6 ± 4.6	0.8
Normal IAP (patients (%))	11 (36.7)	2 (18.2)	0.3
Maximal value of IAP (mmHg, mean ± SD)	14.1 ± 5.6	18.2 ± 6.9	0.06
Minimal value of APP (mmHg, mean ± SD)	80.9 ± 8.8	74.9 ± 7.7	0.05
IAH (patients (%))	19 (63.3)	9 (81.8)	0.3
ACS (patients)	4 (13.3)	5 (45.5)	0.03*
Ileus	23 (76.7)	11 (100)	0.08
Type of ileus (patients (%))			
Impaired gastric emptying	0	0	
Impaired intestinal and colonic emptying	20 (66.7)	1 (9.1)	0.01*
Combined form	10 (33.3)	10 (90.9)	0.01*
Onset time of ileus (postoperative day)	2.6 ± 1.6	1.6 ± 0.7	0.06
Duration of ileus (days)	8.5 ± 6.6	15.3 ± 8.5	0.01*
Risk factors of ileus (patients (%))			
DD	25 (83.3)	8 (72.7)	0.4
Independent risk factors (patients (%))			
Narcotics	0	2 (18.2)	0.02*
Sympathomimetics	12 (40)	9 (81.8)	0.02*
Positive fluid balance	1 (3.3)	6 (54.5)	0.0001*
Sepsis	2 (6.7)	6 (54.5)	0.0006*
Hypokalemia, hypomagnesaemia	19 (63.3)	9 (81.8)	0.3

*Significant difference. SD, standard deviation; APACHE-II, acute physiology and chronic health evaluation, and SOFA, sequential organ failure assessment, scores on ICU admission; IAP, intra-abdominal pressure; APP, abdominal perfusion pressure; IAH, intra-abdominal hypertension; ACS, abdominal compartment syndrome; DD, diencephalon dysfunction.

In survivors, isolated impairment of intestinal/colonic emptying developed in 66.7% of patients; the combination of impaired gastric emptying and impaired intestinal/colonic emptying was revealed in 33.3% of patients. In non-survivors, isolated impairment of intestinal/colonic emptying developed in 9.1% of patients; the combination of impaired gastric emptying and impaired intestinal/colonic emptying was revealed in 90.9% of patients. Therefore, isolated impairment of intestinal/colonic emptying was observed significantly more frequently in group I (*p *= 0.01), whereas the combination of impaired gastric emptying and impaired intestinal/colonic emptying was observed significantly more frequently in group II (*p *= 0.01). Independent risk factors such as positive fluid balance, sympathomimetics, administration of narcotics, and sympathomimetics were revealed significantly more frequently in non-survivors than in survivors. Ileus persisted significantly longer in non-survivors than in survivors, 15.3 ± 8.5 versus 8.5 ± 6.6 days (*p *= 0.01).

All four patients with ACS, who had received EA, survived with favorable outcomes. Three patients with ACS and effective conservative therapy died due to causes independent of IAH (thromboembolism, cardiomyopathy, and sepsis). The other two patients with ACS, who had not received EA, died due to sepsis and meningitis.

## Discussion

A complicated postoperative period developed in 7% of patients with SRT. Two thirds of this neurocritical care population has IAH, which developed on the second postoperative day. IAH correlated with worse APACHE-II and SOFA scores, and it occurred more frequently in non-survivors. Patients with normal IAP had better outcomes. However, the cause-and-effect relationship between IAP and severity of the patients' condition remains unclear. Severe injury to the hypothalamo-pituitary axis can lead to MOD [4]. On the other hand, our patients had high maximal IAP values, and IAH can also be considered as a cause of MOD [5,12]. Our data show the importance of IAP monitoring in these neurocritical care patients since early recognition and timely medical management can affect outcome.

Ileus was a leading cause of IAH in our cohort. Theoretically, capillary leak syndrome and fluid accumulation can also cause IAH [1,3,5]. This syndrome can be due to decompensated hypothyroidism [13] in patients with SRT and can lead to polyserositis with ascites, hydrothorax, and/or hydropericardium [6,14]. In our population, severe hypothyroidism was ruled out because all the patients received postoperative polyhormonal therapy, and the levels of thyroid hormones in plasma were routinely controlled. Also, there were no patients with ascites in our study population.

Different types of ileus were observed: impaired intestinal and colonic emptying and a combined form. The leading cause of ileus was DD, and this can be explained by the surgery site. Hypothalamic nuclei directly regulate gastrointestinal motility [7,15]. Furthermore, a normal thyroid status is necessary for an adequate function of the gastrointestinal tract [13]. Independent risk factors had minor importance for the ileus development in our patients, especially narcotics, sympathomimetics, or positive fluid balance. The last is insignificant for SRT patients because central diabetes insipidus is a common postoperative complication. Sepsis was an important risk factor for ACS. Our results are in concordance with previous literature data [16-18]. Hypokalemia and hypomagnesaemia developed frequently due to glucocorticoid therapy [19].

Conservative treatment was effective in the majority of patients with IAH, but without ACS. In ACS, on the other hand, conservative treatment was ineffective in two thirds of the patients. Guidelines recommend urgent laparotomy if conservative therapy fails [5,8,20]. There are no publications on the timing and efficacy of urgent laparotomy in patients with secondary ACS due to neurosurgical pathology. Thoracic EA, used before laparotomy, should therefore be considered as a safe and effective therapeutic option.

The use of EA is generally accepted in the ICU and has been previously described [10,21,22]. The pathophysiological basis of thoracic EA for the treatment of ACS is: (1) sympathetic block and accordingly, prevalence of parasympathetic tonus in the innervation of the gastrointestinal tract, (2) strong analgetic effect, (3) abdominal wall muscle relaxation, (4) increase of gastrointestinal blood flow, gastric mucosal perfusion, improvement of tissue oxygenation, and (5) prevention of bacterial translocation [22-25]. These mechanisms can interrupt the deleterious pathological processes caused by ACS. We showed that the use of EA can be effective in a selected group of patients. In these patients, the IAP normalized within several hours, and signs of MOD regressed within 1 to 2 days. The duration of IAH in patients with EA was significantly shorter than that in patients without EA. We could not perform EA in two septic patients with ineffective conservative treatment for ACS. Traditionally, sepsis is a contraindication for EA due to an increased risk of infectious complications [21,26]. All patients with ACS, who did not receive EA, died, irrespective of the duration of ACS. These data allow us to conclude the following: (1) early thoracic EA can be an effective treatment option for secondary ACS in neurosurgical patients; and (2) if the main cause of ACS in neurosurgical patients is sepsis, the only effective method of ACS treatment would probably be urgent laparotomy.

To our knowledge, this is the first study to investigate secondary ACS in patients with SRT. Our study has several serious limitations. First, 41 patients are still a small number for a meaningful statistical analysis, especially concerning the calculation of the risk of unfavorable outcomes and death in patients with IAH. Second, it is a single-center study. Third, we only performed intermittent measurements of IAP, not continuous monitoring, which could have changed the therapeutic approach and accordingly, the results. Fourth, four patients with EA are a small number for decision-making about the significance of EA for ACS treatment.

## Conclusions

The development of IAH is common in patients after SRT surgery during a complicated postoperative period. If conservative treatment is ineffective, EA can be attempted in patients with secondary ACS when it is not contraindicated. Further studies are warranted.

## Abbreviations

ACS: abdominal compartment syndrome; DD: diencephalon dysfunction; EA: epidural anesthesia; GOS: Glasgow Outcome Scale; IAP: intra-abdominal pressure; IAH: intra-abdominal hypertension; LOS: length of stay; MOD: multiple organ dysfunction; MV: mechanical ventilation; NICU: neurosurgical intensive care unit; SRT: sellar region tumors.

## Competing interests

The authors declare that they have no competing interests.

## Authors' contributions

PLK, IAS, and AUL contributed to the conception and design of the study. ASG, BAK, and KAP did the acquisition of funding, systematic search, and drafting of the manuscript. AVO, AAP, and EUS did the interpretation of data and drafting of the manuscript. MAK and VIL contributed substantially to the interpretation of data and critically revised the manuscript. All authors read and approved the final manuscript for publication.
